# Comparative efficacy and cytocompatibility of antimicrobial nanocoatings versus antibiotics on titanium dental implant surfaces

**DOI:** 10.6026/973206300223712

**Published:** 2026-06-30

**Authors:** Maitreyi Ranjan, Shailesh Jain, Nikhil Prakash, Monika Sinha, Shabnam Chaudhary, Roli Singh

**Affiliations:** 1 Department of Microbiology, School of Dental Sciences, Sharda University, Greater Noida, Uttar Pradesh, India; 2 Department of Dentistry, GEIMS, Dehradun, Uttarakhand, India; 3 Department of Prosthodontics, Crown and Bridge, DJ College of Dental Sciences and Research, Modinagar, Uttar Pradesh, India; 4 Department of Dentistry, Private Practitioner, Flora Dental care, Bangalore, India; 5 Department of Dentistry, PHC Jahu, Hamirpur, Himachal Pradesh, India; 6 Department of Oral and Maxillofacial Pathology and Microbiology, Visiting consultant, Gupta Dental Care, Aligarh, India

**Keywords:** Titanium dental implants, silver nanoparticles (AgNP), zinc oxide nanoparticles (ZnO-NP), antimicrobial coating, cytocompatibility, peri-implantitis

## Abstract

Bacterial colonization resulting in peri-implantitis is a significant issue in dental implantology which results in an implant failure. Therefore, it is of interest to evaluate the antimicrobial efficacy and cytocompatibility of nanoparticle-based-coatings and conventional antibiotic-coatings on the titanium dental implant surfaces were compared. The determination of the cytocompatibility between human osteoblast-like cells (MG-63) was done in terms of MTT assay, cell proliferation and adhesion. AgNP coating was found to be more effective at antimicrobial efficacy against all bacteria studied (18.6±1.4 mm with S. aureus) than gentamicin (14.2±1.1 mm) and ZnO-NP (12.8±1.3 mm). Thus, data shows the nanoparticle-based coatings especially AgNPs have better antimicrobial effects with retained biocompatibility.

## Background:

The dental implants have transformed the contemporary dentistry to offer predictable and long-term solution to the replacement of teeth with successful success rate of over 95 percent after 10 years [1]. Dental implants continue to be made of titanium and its alloys because they are the most gold standard materials with superior osseointegration, mechanical strength and bio compatibility [2]. Nevertheless, the colonization of implants surface by bacteria, followed by the formation of biofilms is a major complication, which may cause peri-implant mucositis and peri-implantitis, which occurs in about 10-47 per cent of implants [3]. Peri-implantitis is a state of progression of inflammatory responses in the soft and hard tissues around dental implants and eventual bone loss and eventual implant loss [4]. This microbial etiology is characterized by complex polymicrobial biofilms, the majority of which are Staphylococcus aureus, Streptococcus species, Pseudomonas aeruginosa and Escherichia coli [5]. Biofilms once formed show incredible resistance to the traditional antimicrobial treatment options and the preventive therapy techniques based on surface modification should be aimed at preventing the initial bacterial adhesion and colonization [6].Titanium implant surface modification techniques have developed over the years and there are different methods that are used to improve anti-bacterial and the rates of implantation [7]. Traditional approaches involve the antimicrobial coating of implant surfaces with gentamicin, vancomycin, or tetracycline which offers local antimicrobial effects but has low systemic exposure [8]. Nonetheless, implants coated with antibiotics have a number of drawbacks such as limited duration of action, the possibility of bacteria developing resistance to the antibiotic, the possibility of an allergy and cytotoxicity problems to host cells [9]. With the development of nanotechnology, the opportunities of the surface engineering of implants have become available. Metallic nanoparticles, especially silver nanoparticles (AgNPs) and zinc oxide nanoparticles (ZnO-NPs) have shown a high level of broad-spectrum antimicrobial activity via various mechanisms, such as cell membrane disruption, production of reactive oxygen species (ROS) and bacterial DNA replication inhibition [10]. The antimicrobial properties of silver nanoparticles have greatly been explored in biomedical applications because it is an exceptional antimicrobial agar with Gram-positive and Gram-negative bacterial and fungal strains [11]. On the same note ZnO-NPs also have strong antibacterial properties and they have good biocompatibility and photocatalytic properties [12]. Recent investigations have examined the use of nanoparticles to titanium implant surfaces using different methods such as physical vapor deposition, electrochemical deposition and sol-gel [13]. Although the literature has already independently measured either antimicrobial properties or biocompatibility of nanocoatings, there are limited literatures available to directly compare the two parameters against traditional antibiotic coatings [14]. Additionally, the lack of information that compares various nanoparticle systems to conventional antibiotic methods in the same experimental conditions is set to be felt. Antimicrobial effectiveness and cytocompatibility is a critical issue in the design of implant surfaces. A perfect coating is both able to resist bacterial colonization and at the same time facilitate the osteoblast to attach, grow and differentiate in order to facilitate successful ossification [15]. Therefore, it is of interest to determine the antimicrobial efficacy and cytocompatibility of silver nanoparticle and zinc oxide nanoparticle coating on titanium dental implant surfaces.

## Materials and Methods:

## Study design and sample preparation

This was an *in vitro* experimental comparative study conducted at the Department of Dental Materials and Microbiology Research Laboratory over a period of 12 months. Sixty commercially pure grade IV titanium discs (diameter: 10 mm, thickness: 2 mm) were fabricated and polished to a standardized surface roughness (Ra = 0.4 μm) using sequential silicon carbide papers (600-2000 grit). All discs were ultrasonically cleaned in acetone, ethanol and distilled water (15 minutes each), then sterilized by autoclaving at 121°C for 20 minutes.

## Surface coating procedures:

The titanium discs were randomly divided into four groups (n=15 per group):

[1] Group 1 (Control): Uncoated titanium discs serving as negative control.

[2] Group 2 (Gentamicin coating): Discs were coated with gentamicin sulfate (concentration: 2 mg/mL) using a dip-coating technique. Specimens were immersed in gentamicin solution for 24 hours, followed by air-drying in a laminar flow hood.

[3] Group 3 (AgNP coating): Silver nanoparticles (average size: 20-30 nm, spherical morphology) were synthesized using chemical reduction method with silver nitrate and sodium borohydride. Titanium discs were coated via electrophoretic deposition at 20V for 5 minutes, yielding uniform AgNP coverage at concentration of 50 μg/cm^2^.

[4] Group 4 (ZnO-NP coating): Zinc oxide nanoparticles (average size: 30-40 nm) were obtained commercially (Sigma-Aldrich) and applied through sol-gel technique. A ZnO-NP suspension (100 μg/mL in ethanol) was spin-coated onto titanium surfaces at 2000 rpm for 30 seconds, followed by calcination at 400°C for 2 hours.

Surface characterization was performed using scanning electron microscopy (SEM) and energy-dispersive X-ray spectroscopy (EDX) to confirm coating uniformity and composition.

## Bacterial strains and culture conditions:

Three bacterial strains representing common implant-associated pathogens were obtained from American Type Culture Collection (ATCC): Staphylococcus aureus (ATCC 25923), Escherichia coli (ATCC 25922) and Pseudomonas aeruginosa (ATCC 27853). Bacteria were cultured in L uria-Bertani broth at 37°C with continuous shaking (150 rpm) to mid-logarithmic phase (OD^600^ = 0.5, approximately 1x10^8^ CFU/mL).

## Antimicrobial activity assessment:

## Zone of Inhibition (ZOI) Test:

Coated titanium discs were placed on Mueller-Hinton agar plates inoculated with bacterial lawns (1x10^6^ CFU/mL). After 24-hour incubation at 37°C, inhibition zones were measured in millimetres using digital callipers (n=5 per group per bacterium).

## Bacterial adhesion assay:

Discs were placed in 24-well plates containing 1 mL bacterial suspension (1x10^7^ CFU/mL) and incubated for 4 hours at 37°C. Non-adherent bacteria were removed by gentle washing with phosphate-buffered saline (PBS). Adherent bacteria were detached by sonication and vortexing and then quantified by serial dilution and colony counting on agar plates. Results were expressed as CFU/cm^2^ and percentage reduction compared to control.

## Biofilm formation assay:

Titanium discs were incubated with bacterial suspensions for 48 hours. Biofilms were stained with crystal violet (0.1%) and absorbance was measured at 570 nm using a microplate reader.

## Cytocompatibility evaluation:

Human osteoblast-like cells (MG-63 cell line, ATCC CRL-1427) were cultured in Dulbecco's Modified Eagle Medium (DMEM) supplemented with 10% fetal bovine serum and 1% penicillin-streptomycin at 37°C in 5% CO_2_ atmosphere.

## Cell viability assay (MTT):

Cells were seeded on coated titanium discs (1x10^4^ cells/disc) in 24-well plates. After 24, 48 and 72 hours, MTT reagent (0.5 mg/mL) was added and incubated for 4 hours. Formazan crystals were dissolved in dimethyl sulfoxide and absorbance was measured at 570 nm. Cell viability was calculated as percentage relative to control.

## Cell proliferation assay:

Cell proliferation was assessed using BrdU incorporation assay after 72 hours of culture. Results were quantified spectrophotometrically and expressed as proliferation index.

## Cell adhesion and morphology:

After 24-hour culture, cells on titanium surfaces were fixed with 4% paraformaldehyde, permeabilized and stained with DAPI for nuclear visualization and phalloidin-FITC for actin cytoskeleton. Specimens were examined using fluorescence microscopy. Cell adhesion was quantified by counting adherent cells in five random fields per specimen.

## Statistical analysis:

All experiments were performed in triplicate and data were expressed as mean ± standard deviation (SD). Statistical analyses were conducted using SPSS version 24.0 software. One-way analysis of variance (ANOVA) followed by Tukey's post-hoc test was used for multiple group comparisons. Two-way ANOVA was employed for time-dependent analyses. Statistical significance was set at p<0.05.

## Results:

SEM analysis confirmed uniform coating distribution across all treated titanium surfaces. EDX spectroscopy verified the presence of silver and zinc in respective nanoparticle-coated groups, with coating thicknesses ranging from 0.5-1.2 μm for all modified surfaces. Table 1 presents the zone of inhibition results for different coating groups against three bacterial strains. AgNP-coated surfaces demonstrated significantly larger inhibition zones against all tested bacteria compared to other groups (p<0.001). Against S. aureus, AgNP coatings produced zones of 18.6 ±1.4 mm, significantly superior to gentamicin (14.2 ±1.1 mm, p<50.001) and ZnO-NP (12.8 ±1.3 mm, p±0.001). Similar trends were observed for E. coli and P. aeruginosa. Control surfaces showed no inhibition zones. Table 2 shows bacterial adhesion results after 4-hour incubation. AgNP coatings exhibited the highest reduction in bacterial adhesion (85.4% for S. aureus) compared to control surfaces (p<0.001), followed by gentamicin (72.6%) and ZnO-NP (64.8%). All modified surfaces significantly reduced bacterial adhesion compared to control (p<0.001). The CFU counts on control surfaces ranged from 3.8x10^5^ to 5.2x10^5^ CFU/cm^2^, while AgNP surfaces maintained counts below 7.6x10^4^ CFU/cm^2^ for all bacteria tested.Values expressed as Mean ±SD (n=5). Different superscript letters indicate significant differences within columns (p<0.05). All coated surfaces showed significantly lower bacterial adhesion compared to control (p<0.001). Biofilm formation assay demonstrated similar patterns, with AgNP coatings showing 78.4% biofilm reduction, gentamicin 61.2% and ZnO-NP 54.6% compared to control surfaces (all p<0.001). Table 3 presents cell viability results at different time points. Both nanoparticle coatings maintained excellent cytocompatibility with cell viability exceeding 90% at all time points, comparable to control surfaces (p<0.05). In contrast, gentamicin-coated surfaces showed significantly reduced cell viability (76.3±4.2% at 72 hours, p<0.01), indicating moderate cytotoxic effects. Cell proliferation index for AgNP and ZnO-NP groups (0.94±0.07 and 0.92±0.08, respectively) showed no significant difference from control (1.00±0.08, p>0.05), while gentamicin coating significantly suppressed proliferation (0.68±0.06, p<0.01). Cell adhesion density was comparable between control, AgNP and ZnO-NP groups (approximately 135-143 cells/mm^2^), but significantly reduced on gentamicin s urfaces (98.4±10.2 cells/mm^2^, p<0.01). Fluorescence microscopy revealed well-spread osteoblasts with prominent stress fibers and normal morphology on control and nanoparticle-coated surfaces. Cells on gentamicin surfaces exhibited rounded morphology with reduced spreading, indicating compromised adhesion and cytoskeletal organization.

## Discussion:

The current research projects give detailed comparative data that clearly show that nanoparticle-based antimicrobial coating, especially silver nanoparticles, possess a greater antimicrobial activity and a high level of cytocompatibility than traditional antibiotic antimicrobial coatings on titanium dental implant surfaces. These results have important implications on the designing of the next-generation implant systems that have high resistance to the colonization of bacteria without the need to affect the process of osseointegration. The excellent antimicrobial activity of AgNP coatings in our experiment is consistent with the known mechanisms of bacterial killing mediated by nanoparticles. Silver nanoparticles act upon antimicrobial action via several pathways such as membrane disruption, protein denaturation, ROS generation as well as DNA damage [16]. This is a multi-targeted solution which minimizes the chances of developing resistance of the bacteria as opposed to the single-mechanism antibiotics [17]. The penetration ability of the nanoparticles due to their size allows them to react better with the membranes and intracellular structures of the bacteria hence their increased effectiveness against Gram-positive and Gram-negative bacteria as can be seen in our study. The fact that AgNP coating resulted in 85.4% inhibition of bacterial adhesion is in agreement with other studies that have found that titanium surface coated with silver displays high anti-adhesive properties [10]. The initial and most important step in biofilm formation is bacterial adhesion and preventing the early colonization is better than trying to eliminate formed biofilms [18]. The permanence of antimicrobial action of coatings on nanoparticles demonstrated by the lack of diminishing inhibition areas and decreased biofilm formation is beneficial compared to antibiotic coatings, which can lose their ability to combat microbes through depletion and degradation of the drug. The antimicrobial efficacy of ZnO-NP coatings was moderately high, which can be explained by the release of zinc ions and the spatial generation of ROS [12]. ZnO nanoparticles are not as strong as AgNPs but have such advantages as low cost, higher stability and reported osteogenic properties which can be useful in inducing bone formation [15]. The selection of alternative nanoparticle systems can be conditional on the situation of a particular clinical case, cost efficiency and preferred ratio between antimicrobial and osteogenic functions. The cytocompatibility results are a significant part of our research, as they deal with a popular issue of the cytotoxicity of nanoparticles. AgNP and ZnO-NP coating showed cell viability of above 90 and both are excellent biocompatible to titanium in their uncoated form. This is in contrast to some previous reports that have indicated nanoparticle cytotoxicity, and this can be explained by the fact that our methodology of control of coating has resulted into the achievement of optimal nanoparticle concentration and distribution [11]. The nanoparticles that are immobilized on surfaces offer localized antimicrobial action but reduce systemic or excessive local action which may damage host cells. On the other hand, gentamicin coating was highly cytotoxic, decreasing the viability of the osteoblast to 76.3% and affecting cell adhesion and proliferation. The results are similar to those of other studies that have found a dose-dependent cytotoxicity of aminoglycoside antibiotics in osteoblasts [9]. This is a severe weakness of antibiotic coatings the therapeutic window between antimicrobial efficacy and cytotoxicity can be slim and this can inhibit the process of osseointegration, even during infection treatment. Data on quantitative viability is corroborated by the observations of the fluorescence microscopy of normal cell spreading and the organization of cytoskeleton formation on nanoparticle surfaces. Appropriate cell adhesion and cytoskeleton development are needed to be followed by further osteoblastic differentiation and matrix generation that would lead to successful osseointegration [2]. The cell morphology of the maintained cells upon nanocoated surface indicates that these additions do not disrupt normal cellular activities. Clinically, our results may indicate that nanoparticle coating more effectively tackles the twin issues of preventing the implant infections as well as promoting the process of osseointegration compared to the currently used method of using antibiotics. This has been aggravated by rising issues relating to antibiotic resistance; hence, non-antibiotic antimicrobial approaches continue to gain importance [16]. Nanoparticle coating does not promote the development of antimicrobial resistance, which is a long-term solution that is sustainable. There are a number of limitations that should be considered. In this *in vitro* experiment, individual cultures of bacteria have been used, whereas in clinical implant infections, polymicrobial biofilms experience complicated interactions [19, 20]. The efficacy of nanocoating in comparison with mixed-species biofilms in more closely related conditions to the oral environment should be assessed in future research. Also, nanoparticle surface stability over a long period of time, possible kinetics of release of nanoparticles and *in vivo* biocompatibility of nanoparticles need to be studied using animals prior to clinical usage. The contact between nanocore shelled surfaces and other host cells such as immune cells and gingival epithelial cells are also worthy of study [21].

## Conclusion:

Antimicrobial nanocoatings, specifically, silver nanoparticles have better antimicrobial activity against common implant-associated pathogens than gentamicin antibiotic coating and are very cytocompatible with osteoblasts. AgNP and ZnO-NP coating are efficient in reducing the adhesion and the formation of biofilm by bacteria without affecting the cell viability, adhesion and growth. Thus, nanoparticles in surface modifications as viable alternatives to antibiotic coating in peri-implantitis prevention and successful integration of osteosis in dental implantation therapy.

## Figures and Tables

**Table 1:** Zone of inhibition (mm) against bacterial strains

**Surface Coating**	**S. aureus**	**E. coli**	**P. aeruginosa**	**Mean ZOI**
Control	0.0±0.0^a^	0.0±0.0^a^	0.0±0.0^a^	0.0±0.0^a^
Gentamicin	14.2±1.1^b^	12.8±0.9^b^	11.4±1.0^b^	12.8±1.4^b^
AgNP	18.6±1.4^c^	16.9±1.2^c^	15.3±1.1^c^	16.9±1.7^c^
ZnO-NP	12.8±1.3^b^	11.2±1.0^d^	10.6±0.9^b^'^d^	11.5±1.2^b^
Values expressed as Mean±SD (n=5).
Different superscript letters indicate
statistically significant differences within columns (p<0.05,
one-way ANOVA with Tukey's post-hoc test).
AgNP: Silver nanoparticles;
ZnO-NP: Zinc oxide nanoparticles.

**Table 2:** Bacterial adhesion on different titanium surfaces

**Surface**	**S. aureus (CFU/cm^2^ x 10^4^)**	**% Reduction**	**E. coli (CFU/cm^2^ x 10^4^)**	**% Reduction**	**P. aeruginosa (CFU/cm^2^ x 10^4^)**	**% Reduction**
Control	48.6±6.2^a^	-	52.1±7.4^a^	-	38.4±5.8^a^	-
Gentamicin	13.3±2.1^b^	72.60%	16.8±2.4^b^	67.80%	12.2±1.9^b^	68.20%
AgNP	7.1±1.4^c^	85.40%	7.6±1.6^c^	85.40%	6.4±1.2^c^	83.30%
ZnO-NP	17.1±2.8^b^	64.80%	19.4±3.2^b^	62.80%	14.8±2.3^b^	61.50%

**Table 3:** Osteoblast cell viability (%) and proliferation on titanium surfaces

**Surface**	**24 hours**	**48 hours**	**72 hours**	**Proliferation Index**	**Cell Adhesion (cells/mm6^2^)**
Control	100.0±5.2^a^	100.0±6.1^a^	100.0±5.8^a^	1.00±0.08^a^	142.6±12.4^a^
Gentamicin	84.2±5.8^b^	79.6±4.6^b^	76.3±4.2^b^	0.68±0.06^b^	98.4±10.2^b^
AgNP	96.4±4.9^a^	94.2±5.3^a^	92.8±5.6^a^	0.94±0.07^a^	136.8±11.6^a^
ZnO-NP	95.8±5.1^a^	93.6±4.8^a^	91.4±5.2^a^	0.92±0.08^a^	134.2±13.1^a^
Values expressed as mean±SD (n=5).
Cell viability expressed as percentage
relative to control at respective time points.
Different superscript letters indicate
significant differences within columns (p<0.05).
Proliferation index calculated relative to control.
Cell adhesion counted after 24-hour culture.
